# The Real-World Use of Guideline-Directed Medical Therapy in Patients with Heart Failure with Reduced Ejection Fraction Newly Initiated on Dapagliflozin: An EVOLUTION-HF CEE-BA Study (Results from the Baltic Cohort)

**DOI:** 10.3390/medicina62071310

**Published:** 2026-07-07

**Authors:** Diana Žaliaduonytė, Paulius Orda, Olar Pullisaar, Mai Blöndal, Roma Kavaliauskienė, Jolanta Laukaitienė, Natalija Pontaga, Vytė Maneikienė, Aldis Strēlnieks, Kārlis Trušinskis, Tiina Uuetoa, Valters Stirna, Anu Hedman

**Affiliations:** 1 Medical Academy, Lithuanian University of Health Sciences, LT-44307 Kaunas, Lithuania; 2 Klaipeda University Hospital, LT-92288 Klaipeda, Lithuania; 3 North Estonia Medical Centre, 13419 Tallinn, Estonia; 4 Tartu University Clinic, 50406 Tartu, Estonia; 5 Cardiology Clinic, Lithuanian University of Health Sciences, LT-44307 Kaunas, Lithuania; 6 Daugavpils Regional Hospital, LV-5401 Daugavpils, Latvia; 7 Clinic of Cardiac and Vascular Diseases, Vilnius University, LT-03101 Vilnius, Lithuania; 8 Riga East University Hospital, LV-1038 Riga, Latvia; 9 Pauls Stradiņš Clinical University Hospital, LV-1002 Riga, Latvia; 10 Confido Medical Centre, 10151 Tallinn, Estonia; 11 Liepajas Regional Hospital, LV-3401 Liepaja, Latvia; 12 East Tallinn Central Hospital, 10138 Tallinn, Estonia

**Keywords:** heart failure with reduced ejection fraction, dapagliflozin, GDMT, the Baltics, real-world

## Abstract

*Background and Objectives*: Guideline-directed medical therapy (GDMT) remains a challenge due to its low rates worldwide. Adherence to GDMT in patients with heart failure (HF) and reduced ejection fraction (rEF) in the Baltics (Estonia, Latvia, and Lithuania) has been unknown. This is the first study to describe the characteristics of patients with HFrEF and their treatment patterns in the Baltics. *Materials and Methods*: The EVOLUTION-HF study was a longitudinal, real-world evidence study that enrolled patients with HFrEF following newly initiated sodium–glucose cotransporter-2 inhibitor (SGLT2i) dapagliflozin therapy. In total, 12 study sites (four from each country) were included. Patients were followed up for 12 months. A pooled data set including adherence to GDMT, dapagliflozin’s usage, and patients’ profiles was evaluated. *Results*: Between April and December 2022, 178 patients with a median left ventricular ejection fraction of 32% were enrolled. At baseline, 155 (87%) patients received renin–angiotensin–aldosterone system inhibitors (RAASi), 168 (94%) received beta-blockers (BB), and 106 (60%) received mineralocorticoid receptor antagonists (MRA); however, only 47.8% of the study population were found to be treated with GDMT (including RAASi, BB, MRA, and dapagliflozin) at the beginning of the study. At the end of the follow-up, the percentage of GDMT users decreased to 42.3%. The number of dapagliflozin discontinuations per 100 patient-years was found to be 13.2 (95% CI 8.2–18.2). *Conclusions*: The findings indicate a low rate of dapagliflozin discontinuation (13%) and an overall suboptimal proportion of patients receiving GDMT for HFrEF (almost half of the patients). The low rate of GDMT use in the Baltics prompts great attention. Additional research and incremental joint efforts are needed to ensure an increase in GDMT use across diverse HF patient populations.

## 1. Introduction

Heart failure (HF) affects over 64 million people globally, and its burden is expected to increase substantially due to an aging population [1]. HF prevalence varies across Europe, distinguishing the Baltic region from other European countries [2,3]. In Lithuania, HF prevalence accounts for 3.1% in the general population, while systematic epidemiological data from Estonia and Latvia are lacking [2]. The 2019 *European Society of Cardiology (ESC) Atlas* reported that Estonia and Lithuania (no data available for Latvia) had among the highest estimated HF incidence rates in Europe, ranking second and third after Germany, with 6.0 and 4.67 cases per 1000 person-years, respectively [3].

Globally, more than half of the HF population presents with HF with reduced ejection fraction (HFrEF) [4]. Although there has been a substantial reduction in the incidence of HFrEF [4,5], mortality rate ratios have not declined despite recent advances in medical therapy [6]. In Europe, persistent variability in HF care resources and reimbursement may contribute to ongoing outcome gaps [7]. Differences in socioeconomic status, access to medical services, and national health policies may hinder consistent implementation of evidence from clinical trials and guideline-directed medical therapy (GDMT), potentially contributing to variation in cardiovascular morbidity and mortality [2,3,7,8].

Current management of HFrEF requires rapid implementation of GDMT. The ESC guidelines recommend early initiation of the four pillars of HFrEF therapy, including renin–angiotensin–aldosterone system inhibition (RAASi) with an angiotensin-converting enzyme inhibitor (ACE-I), angiotensin-receptor blocker (ARB) or, preferably, an angiotensin receptor–neprilysin inhibitor (ARNI) in eligible patients; an evidence-based beta-blocker (BB); a mineralocorticoid receptor antagonist (MRA); and a sodium–glucose cotransporter-2 inhibitor (SGLT2i, dapagliflozin, or empagliflozin). SGLT2is, developed originally as glucose-lowering agents for the management of type 2 diabetes (T2D), extend their benefit in the cardio–renal spectrum and have transformed the current treatment paradigm for HFrEF, leading to fewer HF worsening events and hospitalizations, and a lower risk of cardiovascular death [9,10].

Despite robust evidence supporting the use of these agents to reduce mortality, improve prognosis, and functional capacity among patients with HFrEF [4,11], real-world use of GDMT in eligible patients remains suboptimal, even low [12,13,14]. Persistent challenges in the strategy implementation and adherence to GDMT create a substantial discrepancy between the demonstrated efficacy of HF interventions in randomized controlled trials (RCTs) and their use in routine medical practice and effectiveness [15]. In addition, real-world data on patient profiles and prescribing patterns across GDMT classes are lacking in many geographies. Gathering such data in a systematic manner could help identify local implementation gaps and inform targeted, context-specific strategies to improve GDMT uptake and optimization.

The global EVOLUTION-HF program aimed to provide real-world evidence on the demographic characteristics and treatment patterns in patients with HFrEF after dapagliflozin was approved for this indication in 2020 [16]. The EVOLUTION-HF multi-country program demonstrated delayed initiation of dapagliflozin and sacubitril/valsartan, frequent discontinuation of ACEi/ARB/BB/MRA, and higher persistence with SGLT2is in Japan, the United States, and Sweden [12], as well as a delay of dapagliflozin initiation of around 23–52 months in Italy, Portugal, and the United Kingdom (UK) [17,18]. In the Central Eastern Europe and Baltic Area (CEE-BA) roll-out of the EVOLUTION-HF program, the following countries participated: Bulgaria, Croatia, Estonia, Hungary, Latvia, Lithuania, Poland, Romania, and Slovenia [19]. We present here demographic characteristics and GDMT treatment patterns in patients with HFrEF from Estonia, Latvia, and Lithuania, which constituted the Baltic cohort. To our knowledge, this is the first study to describe the clinical characteristics of the HFrEF patient population who initiated dapagliflozin as add-on therapy in the Baltic region and to indirectly evaluate GDMT prescription patterns.

## 2. Materials and Methods

### 2.1. Study Design and Population

EVOLUTION-HF was an observational, longitudinal cohort study with a secondary data collection design, conducted in the CEE-BA region between 22 February 2022 and 30 December 2023 [19]. In the Baltic countries, patient enrollment began on 1 April 2022 and ended on 29 December 2022, with the last patient data collection on 12 December 2023.

Adult patients aged 18 years or older with HFrEF (left ventricular ejection fraction [LVEF] ≤ 40%) who were newly initiated on dapagliflozin at the discretion of their healthcare provider, in accordance with the local label, and who provided written consent for data collection were included in this study. Exclusion criteria were as follows: enrollment less than 30 days or more than 60 days following initiation of dapagliflozin, prior treatment with dapagliflozin or other SGLT2i, initiation of dapagliflozin outside of the local HF label, and type 1 diabetes. Given the descriptive nature of this study, a minimum of 150 patients per country (where the three states forming the Baltic Area were considered one single entity) were planned to be enrolled to achieve sufficient precision for key estimates. Assuming a discontinuation rate of around 10% for dapagliflozin, this sample size provides a ±5% margin of error.

The study data were collected by the treating cardiologist or delegated research personnel from both the paper and electronic medical charts of the study participants. The study index date was defined as the date of dapagliflozin initiation for HFrEF. The baseline period was defined as a 12-month interval prior to dapagliflozin initiation. The patients were prospectively followed for up to 12 months after the index date, or until loss to follow-up, death, or study discontinuation, whichever occurred first. The follow-up was performed remotely (by telephone) or at the study site at 6 and 12 months (+/− 1 month). Patient and disease characteristics and other HF and associated T2D treatments, including dose changes for HF medication, were collected. The data were entered by authorized personnel into a secure, pseudonymized web-based electronic case report form. Spontaneous reports of AEs consistent with routine clinical practice followed the local pharmacovigilance requirements but were not collected as part of the study protocol.

This study was conducted in accordance with the Declaration of Helsinki and approved by the Research Ethics Committee of the University of Tartu (UT REC), approval number 356/T-10, approval date 20 December 2021; the Ethics Committee for Clinical Research at Pauls Stradins Clinical University Hospital Development Society, approval number 240522-1E, approval date 24 May 2022; and the Lithuanian Bioethics Committee (Lietuvos Bioetikos Komitetas), approval number L-22-04/1, approval date 28 April 2022.

### 2.2. Study Objectives and Variables

This study had 3 primary objectives [19]: (1) to describe the baseline demographic and clinical characteristics of patients newly prescribed dapagliflozin for the treatment of HFrEF; (2) to assess treatment patterns for dapagliflozin, including time to discontinuation; and (3) to assess treatment patterns for other HF and glucose-lowering medications over time.

Study variables were collected at pre-specified time points of 6 and 12 months since the index date. The baseline data included demographics, selected cardiovascular and non-cardiovascular comorbidities, history of HFrEF and disease characteristics, including ischemic or non-ischemic etiology (according to the investigator’s clinical judgement), and previous pharmacological treatment for HF and T2D, as applicable. The laboratory data (e.g., estimated glomerular filtration rate [eGFR] and N-terminal pro–B-type natriuretic peptide [NT-proBNP]) were collected from the most recent assessment at the index date or within the 6 months prior to the index date, if available. LVEF was collected at the index date. Changes in HF and T2D treatments, including time to discontinuation, initiation of additional medications, discontinuations, and/or dose adjustments, were assessed at 6 and 12 months after the index date.

### 2.3. Statistical Analysis

The full analysis set (FAS) comprised all patients with HFrEF who met eligibility criteria and were included in this study. Variables with a normal distribution were summarized using the mean and standard deviation (SD), while those with a non-normal distribution were summarized by median, interquartile range (IQR), minimum (min), and maximum (max). The categorical data were tabulated as counts and percentages. These variables were descriptively analyzed.

The Kaplan–Meier method was used to estimate the median time to discontinuation (mTTD), with 95% confidence intervals (CIs) for dapagliflozin and for other HF and glucose-lowering medications. The patients who continued treatment for the entire observation period were censored at their last recorded visit. A multivariate regression model (unadjusted Cox proportional hazards) was used to evaluate the association between baseline characteristics and dapagliflozin discontinuation.

Statistical analyses were performed using the R programming language (https://www-r-project.org/) version 4.2.3 (15 March 2023), Shortstop Beagle.

## 3. Results

### 3.1. Study Population and Demographic Characteristics

The Baltic centers enrolled 179 patients with HFrEF, of whom 1 was excluded from analysis for not meeting all of the inclusion criteria. Thus, the FAS included 178 eligible patients: 100 in Estonia, 28 in Latvia, and 50 in Lithuania. The data were collected across 12 centers (four per country), and predominantly hospital-based: 82.6% (147/178) of the patients were treated in hospital centers.

**Figure 1:**
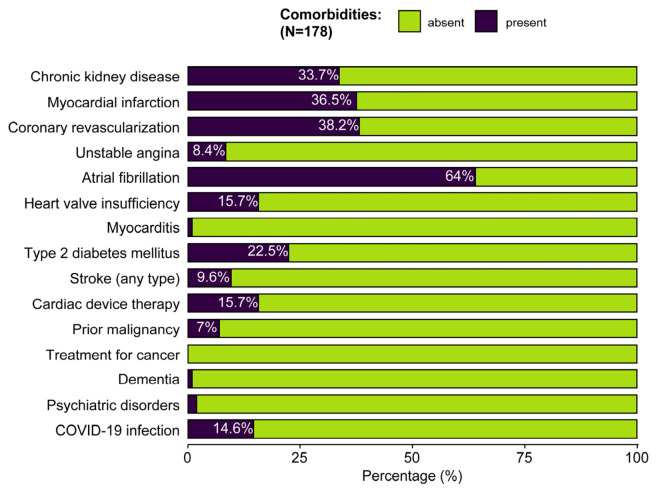
Comorbidities in the study population. Abbreviations: COVID-19, Coronavirus disease, 2019.

Characteristics of the study patients are shown in Table 1. Most of the patients with HFrEF (71.9%) were male, and the median age at the index date was 70.5 years (IQR: 62.0–77.8 years). The median duration of HF at study entry was 22 months (IQR: 1.7–86.0 months), and 32.58% of the patients had a disease duration < 6 months. A proportion of 50.6% of the patients presented with New York Heart Association (NYHA) class II disease, 46.1% with NYHA class III, 2.8% with NYHA IV, and classification was unknown in 1 patient. The most common HF etiology (50.6% of patients) was ischemic heart disease. Half of the patients (50.6%) had three or more comorbidities of interest; atrial fibrillation was the most prevalent (64.0%) concomitant disease (Figure 1). In the year prior to enrollment, 78 (43.8%) of the patients were hospitalized for HF.

### 3.2. GDMT Implementation Outcomes

**Figure 2:**
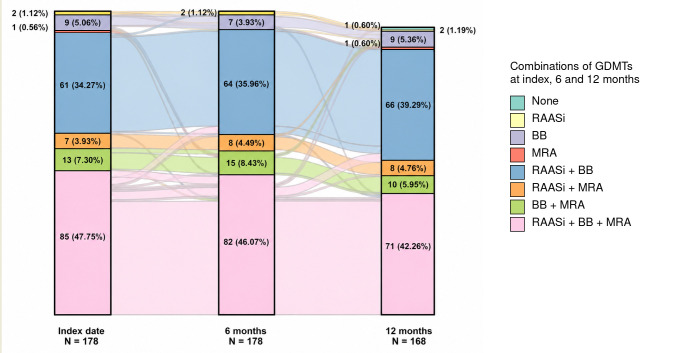
GDMT drug classes at the index date, and 6- and 12-month follow-up. Note: The therapy adjustments depicted in this alluvial plot also include information from patients who discontinued this study (confirmed discontinuations or losses to follow-up). Abbreviations: BB, beta-blockers; GDMT, guideline-directed medical therapy; MRA, mineralocorticoid receptor antagonists; and RAASi, renin–angiotensin–aldosterone system inhibitors.

All of the patients with HFrEF in the FAS received the full daily dose of 10 mg of dapagliflozin. At dapagliflozin treatment initiation, 47.8% of the participants (n = 85) were receiving the core GDMT triad (RAASi+BB+MRA) (Figure 2). Among these, the most commonly used were BBs (94.4%), followed by RAASis (87.1%), and MRAs (59.6%).

The percentage of patients using GDMT remained stable at 46.1% (n = 82) at the 6-month follow-up and declined to 42.3% (n = 71) at the 12-month follow-up (Figure 2). For other single or paired drug classes, a small proportion of changes were observed during the 1-year follow-up (Figure 2). Dose adjustments, additions, or discontinuations in RAASis, BBs, and MRA are shown in Table 2.

Discontinuations were recorded in six of the 174 patients (3.5%) for BBs, 13 of 111 (11.7%) for MRAs and for RAASIs, 14 of 83 patients (16.9%) for ACEi, two of 16 (12.5%) for ARB, and three of 75 (4.0%) for ARNIs. mTDD was not reached for any of these classes.

GDMT with concomitant diuretics was used by 37.1% of the patients at the index date (n = 66), and declined to 34.3% at 6 months (n = 61) and 30.4% at 12 months (n = 51).

Dapagliflozin was interrupted by 11 (6.2%) patients between the index date and the 6-month follow-up, and nine (5.4%) between the 6- and 12-month follow-up. Two patients (1.1%) had two interruptions, 16 (9.0%) had one interruption, and the number of interruptions was unknown in nine (5.1%). Eleven patients resumed the therapy during the study period.

In total, 23 patients (12.9%) discontinued dapagliflozin, corresponding to an incidence rate of 13.2 per 100 patient-years (95% CI: 8.2–18.2). mTTD was not reached. Ten patients (5.6%) discontinued it between the index date and the 6-month follow-up, and 13 (7.7%) between the 6- and 12-month follow-up. Reasons for discontinuation of dapagliflozin were not collected during this study.

In an unadjusted Cox proportional hazard regression model, a higher NYHA class and the presence of chronic kidney disease were the strongest factors associated with dapagliflozin discontinuation in this dataset. Other patient characteristics associated with dapagliflozin discontinuation are shown in Table 3.

## 4. Discussion

To our knowledge, EVOLUTION-HF is the first study from the Baltic region to characterize HFrEF patient characteristics and real-world use of dapagliflozin and other agents within GDMT. Despite the proven benefits of quadruple therapy, uptake of RASSI+BB+MRA in addition to dapagliflozin was modest and declined over time, from 47.8% of the patients at the index date (dapagliflozin initiation) to 42.3% after 12 months of follow-up. This finding could explain the cardiovascular outcomes observed in this region. Suboptimal initiation and persistence of GDMT may contribute to the higher cardiovascular mortality observed in Estonia, Latvia, and Lithuania compared with many other European countries [20].

In the Baltic cohort, the patients initiating dapagliflozin were older (mean age 69 years) and predominantly male (71.9%), with ischemic heart disease as the leading HF etiology (50.5% of patients) and atrial fibrillation (64.0%) as the most common cardiovascular comorbidity. This clinical profile is broadly consistent with the other cohorts in the EVOLUTION-HF study (except for the leading HF etiology, which was non-ischemic in the Slovenian, Portuguese, and UK cohorts) and with other real-world HFrEF populations [17,18,21,22,23,24,25,26]. Of clinical interest, sex differences in HFrEF have been described recently, showing that female patients are usually older at diagnosis, have a higher prevalence of HF de novo, and a shorter progression time of the disease [27], yet over time, prognosis in HF hospitalizations and mortality is similar to that of male patients.

Markers of higher clinical risk at dapagliflozin initiation (mean LVEF 30.8%, mean NT-proBNP 3667.1 pg/mL, and 43.8% of the patients hospitalized for HF in the year prior to enrollment) were observed in the Baltic cohort compared with other EVOLUTION-HF cohorts, which reported lower mean LVEF (e.g., Italy 31.9% and Portugal 29.1%) [18,21], lower NT-proBNP (e.g., Slovenia 607.3 pg/mL) [26], and fewer prior-year HF hospitalizations (17.5–33.3%) [18,21,25,26]. Despite this, only 47.8% of the patients received the RAASi+BB+MRA triad at the time of dapagliflozin initiation, a substantially lower proportion than in Italy (66.4%), Slovenia (74.4%), and the CEE-BA cohort (59.9%), but higher than in Bulgaria (35.3%) [18,19,21,25,26]. This pattern suggests that regional differences in GDMT implementation may be influenced less by patient demographics and more by system-level factors (e.g., access, reimbursement, and care pathways) and local prescribing practices. Real-world use of GDMT since initial prescription, dose titration, and time to reach evidence-based target doses, factors that delay reaching the target doses, and treatment effect over time represent important clinical questions to be addressed in future studies across various geographies.

A previous European HF registry suggested that clinical factors, such as drug intolerance or contraindications, are among the main contributors to GDMT non-prescription. However, the proportion of patients not treated specifically due to intolerance/contraindications was relatively small, accounting for 3.2% for RAASis, 2.3% for BBs, and 5.4% for MRAs, and fewer than 33% of the patients received guideline-recommended target doses [28]. Consistent with these gaps, the REPORT-HF registry revealed that only ~33% of hospitalized patients with HF were discharged on triple GDMT (RAASi+BB+MRA); even in high-income countries, the proportion was only 41% at a time when SGLT2i were still under investigation [29]. Other European registries reported higher uptake among selected populations: 65.4% of the patients with LVEF < 35% received RAASi+BB+MRA in the ESC LT registry [30], and 89%, 92%, and 78% of patients received RAASis, BBs, and MRAs, respectively, following an acute HF event or outpatient visit in the ESC EORP HF III registry [31]. In contrast, more recent real-world data highlight persistent implementation gaps. A Swedish study conducted in a primary healthcare setting showed no appreciable increase in the use of quadruple GDMT 2 years following publication of the ESC 2021 HF guidelines, with only ~20% of patients being prescribed the recommended regimen for HFrEF [13]. A Danish study also showed that nearly 50% of patients did not initiate a fourth foundational HF therapy [14]. Similarly, in our cohort, 47.8% of the patients with HFrEF received quadruple therapy. Moreover, adherence to RAASi+BB+MRA declined progressively over 1 year of follow-up, despite scheduled remote telephone contacts with a dedicated cardiologist. Although such contact might be expected to facilitate treatment optimization and timely referral for specialized HF care, our findings suggest that it was insufficient to overcome barriers to sustained GDMT use. Multiple factors may contribute to this, including clinical inertia, patient-related limitations (advanced age or frailty), heterogeneity in the local health care system and clinical practice, and the lack of remote GDMT up-titration strategies, which may impact the prescription of RAASi, BBs, and MRAs [32].

Sociodemographic patient characteristics may also influence GDMT prescribing, although a recent study identified age as the only independent predictor of under-prescription among other factors (i.e., sex, marital status, nationality, place of residence, and educational level) [33]. From a health care provider perspective, differences in awareness and familiarity with evolving evidence may further contribute to the lag between guideline publication and real-world implementation [2,34,35]. GDMT prescription is strongly influenced by the doctor’s decision/willingness, whereas medication adherence is influenced by multiple factors: system-related (reimbursement policy, healthcare delivery, and costs), socioeconomic (income, education, and support), environmental (cultural, religious, family, and social), patient-related (awareness, beliefs, demographics, and mental health), therapy-related (complexity, side effects, and polypharmacy), and condition-related (symptoms and comorbidities) [15,32]. Important for clinicians, other studies have indicated that non-adherence to GDMT has been associated with negative outcomes: higher risks of emergency department visits, hospitalization, and mortality [36,37].

A notably high proportion (64%) of the patients in the Baltic cohort had atrial fibrillation (AF), which exceeds rates typically reported in HFrEF populations. Across other EVOLUTION HF cohorts, AF prevalence ranged between 24% and 46% [17,18,21,25,26], and in the ESC HF registry, AF was reported in 27% of patients with HFrEF [38]. The elevated burden of AF in our study may partly be explained by case mix, as most of the participants were treated in hospital-based centers, where patients tend to have greater disease severity and comorbidity. However, the available evidence suggests that AF itself is unlikely to account for the lower GDMT observed: in the STRONG-HF trial, AF did not impact up-titration in patients recently hospitalized for acute HF, irrespective of LVEF values [39], and the GUIDE-IT trial similarly found no major impact of AF on GDMT prescribing [40]. Based on these results, it is unlikely that the high prevalence of AF in our study could explain the low rates of RAASi+BB+MRA. AF might have affected LVEF evaluation, potentially influencing the classification of patients as HFrEF and subsequently the false GDMT prescription. As LVEF was not reassessed during follow-up in our study, misclassification over time cannot be excluded and should be considered when interpreting treatment patterns.

Dapagliflozin was generally well tolerated in the Baltic cohort, with a low discontinuation rate (12.9%) during 1 year of follow-up, relatively comparable to other EVOLUTION-HF cohorts, including CEE-BA, Portuguese, Bulgarian, and Slovenian cohorts in the EVOLUTION-HF (5–10%) [17,19,21,25,26], and lower than in the Japanese, Swedish, and the United States cohorts (23.5%) [12]. Persistence with dapagliflozin was largely consistent with that observed for other GDMT classes in our study, despite the lack of reimbursement in Latvia and Lithuania during the study period. Chronic kidney disease was the strongest factor associated with dapagliflozin discontinuation, consistent with prior evidence linking worsening of renal function to non-prescription or discontinuation of GDMT [41]. However, since our study was conducted, more clinical experience has been accumulated with the use of dapagliflozin in CKD, and such findings need to be reassessed within the current cardiovascular–kidney–metabolic continuum.

Several GDMT implementation strategies have been proposed. Building on the observation that standard follow-up alone by cardiologists may be insufficient to maintain GDMT use, current evidence suggests that structured remote optimization pathways can improve implementation. One study showed that a multidisciplinary GDMT optimization program, including remote patient monitoring through telehealth by a trained nurse, pharmacist consultation, and close collaboration with a cardiologist, improved GDMT prescribing and was associated with fewer HF admissions [42]. Similarly, a pilot study using wireless device data transmitted to physicians and nurses every 2–4 weeks during medication up-titration showed that remote titration clinics combined with remote monitoring may improve implementation of GDMT in HFrEF [43]. A more recent study from 2025 explored in more depth the effect of the HF nurse-led clinics on drug titration and cardiovascular events, showing a significantly increased rate of GDMT prescription, which led to reduced mortality [44]. These data are convergent and prove how patient monitoring programs could truly benefit from the joint collaboration between nurses and physicians.

Further, detailed analyses are needed to better understand country- and center-level care pathways across the Baltic region, where access to HF-focused services varies across Estonia, Latvia, and Lithuania. For example, Lithuania has the highest density of cardiologists (167.3 per million persons), followed by Estonia (100.4) and Latvia (72.7) [45]. During the study period, differences in system capacity were reported across these countries. Estonia and Lithuania had higher densities of dedicated HF centers (3.8 and 2.8 per million persons, respectively), with multidisciplinary teams involved in both outpatient and inpatient HF care, compared with Latvia (0.5 per million persons) [7], where HF care was typically delivered in general outpatient services. Access to ultrafiltration in cardiology clinics was higher in Estonia (3.8 centers per million persons) and Latvia (2.7) compared with Lithuania (0.7) [7]. Reimbursement policies also varied: ACEis/ARBs/ARNIs, BBs, and MRAs were reimbursed at 75% in Latvia and 75–90% in Estonia, versus 100% in Lithuania [7], with no subsequent changes in HF-related treatment policies or local guidelines. Access to diagnostics likewise varied, with NT-proBNP testing in primary care reimbursed in Estonia and Lithuania for several years, while in Latvia, it has only been reimbursed since 2025. Although comparable Baltic data on rural–urban differences in care levels (primary versus secondary versus tertiary) are lacking, evidence from other settings suggests that such differences may influence access to GDMT [46,47,48,49,50]. A better understanding of these differences in care may enable the implementation of appropriate strategies to reduce regional disparities in outcomes.

This study has some limitations. Firstly, it is a secondary data collection study; thus, some data may be imprecise and limit the interpretation of the results. Early prescriptions for dapagliflozin may have been selectively used in patients with more severe HF or those who did not respond to standard care, as dapagliflozin was not reimbursed in two of the three Baltic countries at the time of this study. The lack of dapagliflozin reimbursement also impacted the target number of patients in Latvia, which has been reduced. Since only patients recently initiated on dapagliflozin were included in this study, this limits the extrapolation of our findings to the entire SGLT2i class. Data on drug intolerance or physician-related reasons for not prescribing GDMT were not collected, although such data could help partially understand the low numbers of GDMT. Self-reporting of drug intake (the method of data collection in our study) is known to overestimate adherence; thus, actual adherence to GDMT could be expected to be lower [15]. LVEF status and NT-proBNP levels were allowed to be collected at the index date or within the past 6 months before patient enrollment, with no follow-up during the 1-year study period. Both variables may change substantially over time and impact treatment decisions. No data were collected on HF hospitalizations or readmissions, which limits the evaluation of treatment impact on morbidity and mortality.

## 5. Conclusions

The present study provides real-world evidence on the clinical features, GDMT treatment patterns, and discontinuation rates among patients with HFrEF starting dapagliflozin in the Baltic region. While discontinuation of dapagliflozin was reduced, the proportion of patients receiving GDMT for HFrEF remains suboptimal (less than 1 in 2), despite a high-risk clinical profile and significant comorbidity burden. This finding prompts great attention, requiring additional research and incremental efforts targeted at increasing the GDMT use across diverse HF patient populations.

## Figures and Tables

**Table 1:** HFrEF patient profiles following newly initiated dapagliflozin in the Baltic cohort.

Baseline Characteristics	Full Analysis Set (N = 178)
Male, n (%)	128 (71.9)
Age at index date, years	
Mean (SD)	692 (11.7)
Median (IQR)	70.5 (62.0–77.8)
LVEF, %	
Mean (SD)	30.84 (7.2)
Median (IQR)	32 (25–37)
Principal cause of HF, n (%)	
Ischemic	90 (50.6)
Non-ischemic	80 (44.9)
Unknown	8 (4.5)
NTpro-BNP, pg/mL (n = 138)	
Mean (SD)	3667.1 (4652.4)
Median (IQR)	2169 (707–4500)
eGFR, mL/min/1.73 m^2^ (n = 150)	
Mean (SD)	65.5 (19.2)
Median (IQR)	64.4 (52.3–80.0)

Abbreviations: N, number of patients in the full analysis set; n, number of patients with data available for a specific parameter; eGFR, the estimated glomerular filtration rate; HF, heart failure; IQR, interquartile interval; LVEF, the left ventricular ejection fraction; NT-proBNP, N-terminal pro–B-type natriuretic peptide; SD, the standard deviation.

**Table 2:** Summary of HF GDMT changes excluding dapagliflozin at 6- and 12-month follow-up in FAS (class level).

	Index to 6 Months	6 to 12 Months
	n (%)	n (%)
ACEi overall changes	N = 15	N = 10
Discontinuation	10 (66.7%)	3 (30.0%)
Dose decrease	1 (6.7%)	3 (30.0%)
Addition	2 (13.3%)	3 (30.0%)
Addition; discontinuation	2 (13.3%) *	1 (10.0%)
ARB overall changes	N = 2	N = 1
Discontinuation	2 (100%)	1 (100%) **
ARNI overall changes	N = 18	N = 11
Addition	11 (61.1%)	1 (9.1%)
Addition and increase	1 (5.6%)	-
Dose decrease	4 (22.2%)	3 (27.3%)
Dose increase	2 (11.1%)	4 (36.4%)
Discontinuation	-	3 (27.3%)
BB overall changes	N = 23	N = 15
Discontinuation	2 (8.7%)	4 (26.7%)
Dose decrease	11 (47.8%)	3 (20.0%)
Dose increase	6 (26.1%)	6 (40.0%)
Addition	4 (17.4%)	2 (13.33%)
MRA overall changes	N = 13	N = 14
Discontinuation	4 (30.8%)	9 (64.3%)
Dose decrease	3 (23.1%)	2 (14.3%)
Dose increase	3 (23.1%)	1 (7.1%)
Addition	3 (23.1%)	2 (14.3%)

Note: For each class, the number of patients on that medication at 6- and 12-month follow-ups is shown. * addition only; and ** or lost to follow-up. Abbreviations: ACEi, angiotensin-converting enzyme inhibitors; ARB, angiotensin receptor blockers; ARNI, angiotensin receptor–neprilysin inhibitor; BB, beta-blockers; FAS, full analysis set; GDMT, guideline-directed medical therapy; MRA, mineralocorticoid receptor antagonists; N, total number of patients; n (%), number (percentage) of patients in a given category.

**Table 3:** Association of baseline characteristics and discontinuation of dapagliflozin. Unadjusted Cox proportional hazard regression model.

Baseline Characteristics	Levels	HR (95% CI)	*p*-Value
**eGFR (mL/min/1.73 m^2^)**		**0.96 (0.93–0.99)**	**0.002**
**NYHA functional class**	II (Ref)		
	III	0.82 (0.34–2)	0.665
	**IV**	**7.08 (1.97–25.46)**	**0.003**
**Age (years) at HF diagnosis**		**1.06 (1.01–1.1)**	**0.008**
**Chronic kidney disease**		**2.81 (1.23–6.42)**	**0.014**
**Heart rate (bpm)**		**1.03 (1–1.05)**	**0.029**
**HF duration (months)**		**0.91 (0.84–0.99)**	**0.032**
**Sex**	Female (Ref)		
	**Male**	**0.43 (0.19–0.97)**	**0.043**
**Age (years) at initiation of** **dapagliflozin therapy**		**1.04 (1–1.09)**	**0.049**
**Age (years) at study entry**		**1.04 (1–1.09)**	**0.049**
HF-related hospitalization		2.24 (0.97–5.21)	0.060
Diastolic blood pressure (mmHg)		0.97 (0.94–1.01)	0.169
Age class	<65 (Ref)		
	65–75	1.42 (0.45–4.46)	0.553
	>75	2.07 (0.72–5.98)	0.180
ARNI SAC/VAL treatments		0.51 (0.19–1.38)	0.187
LVEF (%)		0.14 (0.01–3.39)	0.224
Principal cause of HF	Ischemic		
	Non-ischemic	1.19 (0.51–2.76)	0.685
	Unknown	1.11 (0.14–8.61)	0.923

Note: Bold font indicates statistically significant associations. Abbreviations: ARNI SAC/VAL, angiotensin receptor/neprilysin inhibitor sacubitril/valsartan; bpm, beats per minute; CIs, confidence intervals; eGFR, estimated glomerular filtration rate; HF, heart failure; HR, hazard ratio; LVEF, left ventricular ejection fraction; NYHA, New York Heart Association (Functional Classification for HF).

## Data Availability

The datasets used and/or analyzed during the current study are available from the corresponding author upon reasonable request. Data underlying the findings described in this manuscript may be obtained in accordance with AstraZeneca’s data sharing policy described at https://astrazenecagrouptrials.pharmacm.com/ST/Submission/Disclosure (accessed on 4 March 2026).

## Abbreviations

The following abbreviations are used in this manuscript:

ACEi	Angiotensin-converting enzyme inhibitor
AF	Atrial fibrillation
ARB	Angiotensin receptor blocker
BB	Beta-blocker
bpm	Beats per minute
CEE-BA	Central Eastern Europe and Baltic Area
CI	Confidence interval
COVID-19	Coronavirus disease 2019
eGFR	Estimated glomerular filtration rate
ESC	European Society of Cardiology
FAS	Full analysis set
GDMT	Guideline-directed medical therapy
HF	Heart failure
HFrEF	Heart failure with reduced ejection fraction
IQR	Interquartile range
LVEF	Left ventricular ejection fraction
MRA	Mineralocorticoid receptor antagonist
mTTD	Median time to discontinuation
NT-proBNP	N-terminal pro-B-type natriuretic peptide
NYHA	New York Heart Association
RAASi	Renin–angiotensin–aldosterone system inhibitor
SD	Standard deviation
SGLT2i	Sodium–glucose cotransporter-2 inhibitor
T2D	Type 2 diabetes
UK	The United Kingdom
